# IgA nephropathy with monotypic IgA-κ deposits: a case report and literature review

**DOI:** 10.3389/fmed.2025.1643630

**Published:** 2025-10-21

**Authors:** Xiao-Ying Li, Guang-Liang Xie, Bo Chen, Ji Zhang, Jian-Sheng Chen, Xiao-Kai Ding

**Affiliations:** ^1^Department of Nephrology, Ruian Hospital of Traditional Chinese Medicine, Ruian, China; ^2^Department of Nephrology, Yueyang Hospital of Integrated Traditional Chinese Medicine and Western Medicine, Shanghai University of Traditional Chinese Medicine, Shanghai, China; ^3^Department of Nephrology, The First Affiliated Hospital of Wenzhou Medical University, Wenzhou, China

**Keywords:** IgA nephropathy, monotypic IgA-κ, mesangial proliferative glomerulonephritis, monoclonal immunoglobulin, case report

## Abstract

IgA nephropathy (IgAN) is an immune complex-mediated glomerulonephritis characterized by predominant IgA deposition in the mesangial region, typically exhibiting polyclonal IgA deposits (co-dominance of κ and λ light chains). However, a few studies have reported IgAN cases with monotypic IgA deposition in glomeruli on renal immunofluorescence, predominantly IgA-λ, while IgA-κ deposition is rare. The pathogenesis, pathological features, and prognosis of IgA-κ monotypic deposition remain poorly understood. Here, we report a case of IgAN with monotypic IgA-κ deposits. The patient presented with microscopic hematuria and non-nephrotic range proteinuria and normal renal function. The renal histopathology revealed mild mesangial hypercellularity with segmental endocapillary proliferation. Both frozen and paraffin immunofluorescence showed monotypic IgA-κ deposition and C3 clumps deposition in mesangial region. Electron microscopy showed electron dense deposition in mesangial region, but no abnormal deposition of monoclonal light chain was observed by immunoelectron microscopy. After 12 months of follow-up, the patient was treated with maximal tolerated doses of renin-angiotensin system inhibitors combined with Nefecon, the patient’s urine protein decreased significantly and renal function was stable, and no hematological disorders were found during the follow-up. Therefore, IgAN with monotypic IgA-κ deposits shares similar clinicopathological features and prognosis with IgAN with polyclonal IgA deposits, suggesting that they may belong to the same disease spectrum. Moreover, IgAN with monotypic IgA-κ deposits and proliferative glomerulonephritis with monoclonal IgA deposits (PGNMID) share similarities in pathological manifestations. Therefore, rigorous monitoring of hematological indices in IgAN patients with monotypic IgA-κ deposits is essential to remain vigilant against misdiagnosis or missed diagnosis of early-stage PGNMID.

## Introduction

IgA nephropathy (IgAN) is the most common form of glomerulonephritis worldwide, characterized pathologically by diffuse deposition of IgA-dominant immune complexes in the glomerular mesangial region (1). Typically, mesangial IgA deposits in IgAN are polyclonal (co-dominant for κ and λ light chains). However, renal immunofluorescence findings in 0–42% of IgAN cases demonstrate monotypic IgA deposits, predominantly IgA-λ (2, 3). This may be attributed to the abundance of B cells producing Gd-IgA1-λ in mucosa-associated lymphoid tissue (MALT) (4), as well as the higher affinity of negatively charged λ light chains for mesangial tissue (5). In contrast, IgAN with monotypic IgA-κ deposits is relatively rare, and its pathogenesis, pathological characteristics, and prognosis remain poorly understood. Herein, we present the clinicopathological features, treatment, and follow-up of a patient with monotypic IgA-κ deposits. This case highlights the importance of improving recognition of this entity, formulating optimal therapeutic strategies, and enhancing patient prognosis. The details are reported as follows.

## Case presentation

A 41-year-old female, with a height of 160 cm and a weight of 65 kg, and no history of chronic diseases, was found to have urinary protein (+) and occult blood (3+) in a routine physical examination 5 months prior to admission. She reported no symptoms such as rash, joint pain, or other discomforts and therefore did not consider these findings significant or seek further medical treatment. One month before admission, laboratory tests showed urine protein (2+), red blood cells (RBC) 170/μL, and white blood cells (WBC) 20/μL, and losartan potassium 100 mg/day was given oral therapy. One day before admission, the patient came to the hospital with “upper respiratory tract infection for 2 days” and urine routine examination indicated protein (+) and red blood cell 22/μL. To further investigate the etiology, the patient was admitted the following day. The blood pressure on admission was 105/68 mmHg, and there was no edema in both lower limbs. The results of laboratory examination showed that hemoglobin was 130 g/L, white blood cells was 5.87 × 10^9^/L, platelets was 302 × 10^9^/L, serum creatinine was 55 μmol/L, eGFR112.2 mL/min/1.73 m2, total blood protein was 67.5 g/L, serum albumin was 37.4 g/L, 24-h urinary protein was 1.42 g, urinary albumin/creatinine was 738 mg/g, urinary protein (++), urinary red blood cell was 170/μL, and immunoglobulin (Ig) A 3.48 g/L, IgG 10.56 g/L, IgM 1.04 g/L, complement C3 1.32 g/L, complement C4 0.28 g/L. Blood free light chain: κ 18.4 mg/L, λ 19.1 mg/L, κ/λ 0.96. ANA 1:100 weakly positive (nucleolar type), ds-DNA, ANCA, anti-GMB antibody, rheumatoid factor (RF) and anti-streptolysin O (ASO) tests were negative, and no monoclonal immunoglobulin was found in blood and urine immunofixation electrophoresis.

**Figure 1:**
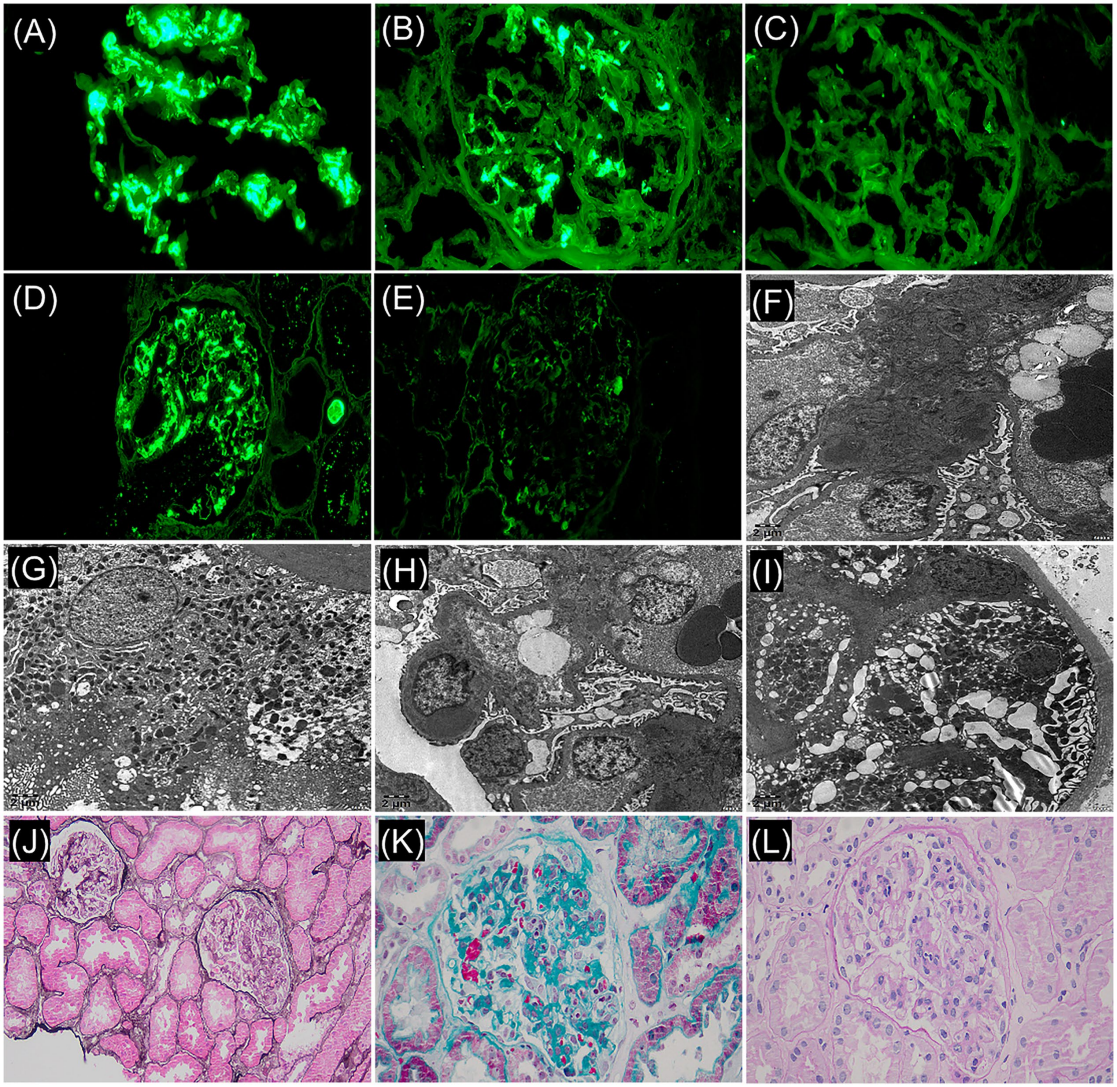
Frozen immunofluorescence (F-IF: ×400): **(A)** IgA: 4+, diffuse, clumpy deposition. **(B)** κ light chain: 4+, diffuse, globular/small mass deposition within the mesangial region. **(C)** λ light chain: negative. Paraffin immunofluorescence (P-IF: ×400): **(D)** κ light chain: 3+, diffuse, globular/ small mass deposition within the mesangial region. **(E)** λ light chain: negative. Immunoelectron microscope (×5,000): **(F)** κ light chain labeling: electron dense matter in mesangia, (+/−). **(G)** κ positive control. **(H)** λ light chain labeling: electron dense matter in mesangial region, (+/−). **(I)** λ positive control. Light microscopy revealed focal hypercellularity within the glomerular capillaries: **(J)** periodic acid-methenamine silver stain + hematoxylin-eosin stain (×200). **(K)** Masson’s trichrome stain (×400). **(L)** Periodic acid–Schiff stain (×400).

To further clarify the etiological diagnosis, a renal biopsy was performed 2 days after admission. The renal biopsy procedure was performed smoothly, and the patient showed no significant discomfort during the process. The pathological findings revealed the following: Renal pathology—light microscopy demonstrated 33 glomeruli, with some showing mild global or segmental mesangial hypercellularity and mild global or segmental mesangial matrix expansion. A few glomeruli exhibited segmental moderate-to-severe endocapillary hypercellularity accompanied by narrowed or occluded capillary lumina, and small cellular-fibrous crescents were observed in 2 glomeruli. Additionally, 5–10% interstitial fibrosis and tubular atrophy were noted, with focal lymphomononuclear cell infiltration and granular or vacuolar degeneration in some tubular epithelial cells. Mild segmental intimal fibrosis was identified in focal interlobular arteries. Immunofluorescence showed IgG (+/− ~+), IgA (4+), IgM (2+), C3 (3–4+), and κ light chain (4+) with diffuse glomerular mesangial granular deposits, while C4, C1q, fibrinogen, and λ light chain were negative. Paraffin immunofluorescence confirmed κ light chain (3+) diffuse glomerular mesangial granular deposits and negative λ light chain. Electron microscopy revealed electron-dense deposits in the glomerular mesangial regions, and immunoelectron microscopy detected no definitive monoclonal light chain deposition (Figure 1). Diagnoses include: (1) IgA nephropathy, focal segmental proliferative glomerulonephritis (Oxford classification: M0 E1 S0 T0 C1). (2) Monotypic κ light chain deposition in glomeruli.

**Figure 2:**
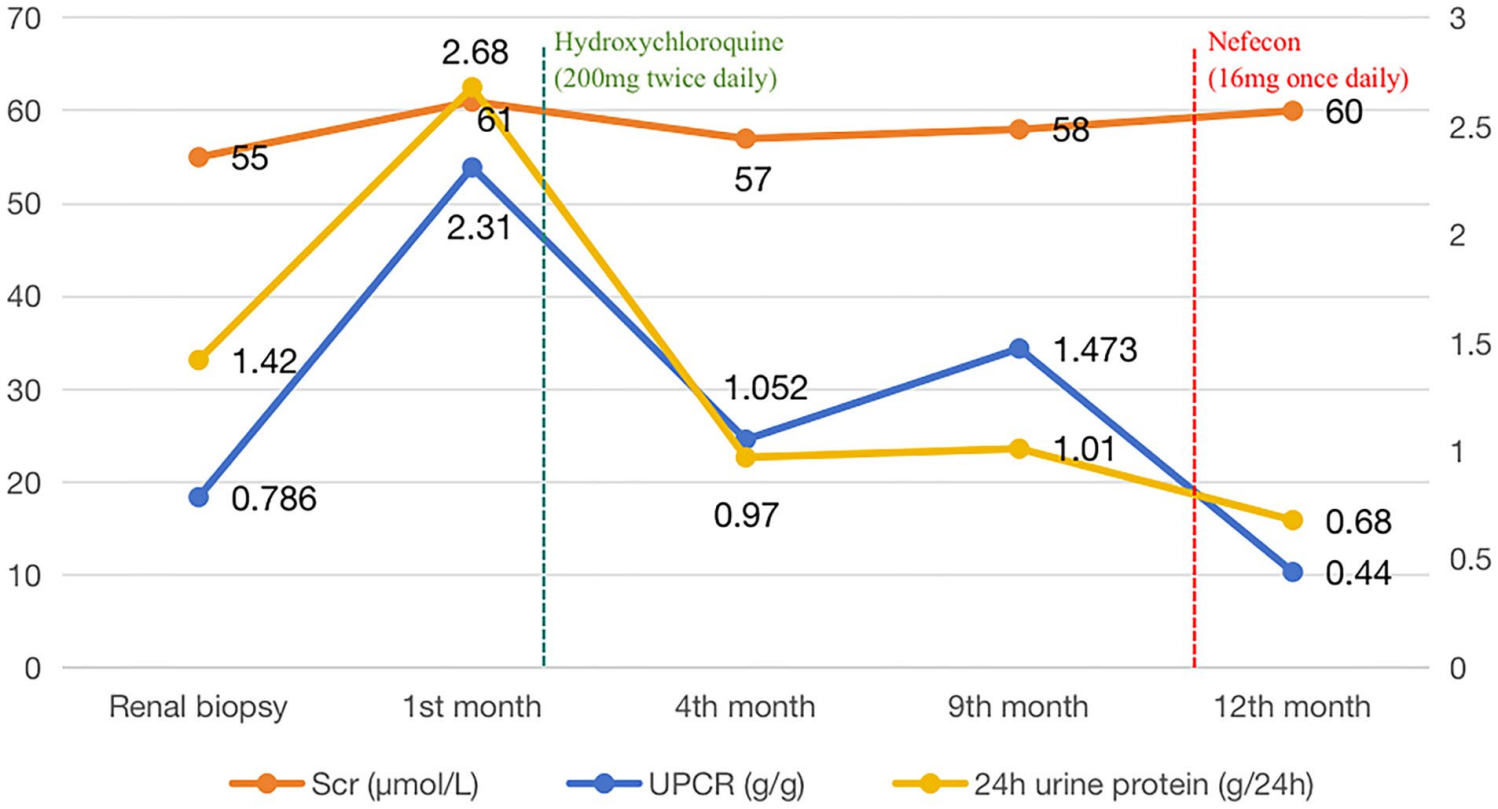
Changes in serum creatinine and urinary protein levels of patients during the follow-up period.

The patient was discharged after a 4-day hospitalization with a total follow-up period of over 12 months. Post-discharge management included continued losartan potassium 100 mg daily for proteinuria control. Follow-up evaluation at 45 days revealed: urine protein-to-creatinine ratio (UPCR) was 1.753 g/g, urine albumin-to-creatinine ratio (UACR) was 1212.7 mg/g. Based on these findings, adjunctive therapy with hydroxychloroquine (200 mg twice daily) was initiated for immunomodulation, maintained for 9 months. The changes of urinary protein and renal function were reviewed repeatedly during follow-up. The 24-h urinary protein of the patient was 0.69–1.01 g, urinary protein/creatinine (UPCR) was 0.95–1.49 g/g, urinary albumin/creatinine (UACR) was 664.5–922.7 mg/g, and blood creatinine was 55–61 μmol/L. eGFR 108.5–112.2 mL/min/1.73 m^2^. During the follow-up period, serum immunofixed electrophoresis was repeated once, but no monoclonal immunoglobulin was detected, and the serum free light chain ratio was 0.81. At the 11-month follow-up, laboratory tests revealed: UPCR 1.49 g/g, UACR 905.8 mg/g, serum creatinine 57 μmol/L. The patient reported that frothy urine remains noticeable and expressed significant anxiety, hoping to receive more effective treatment options. Then Nefecon (16 mg once daily) was added on the basis of the previous treatment. After 51 days of Nefecon treatment, reassessment revealed 24-h urine protein 0.68 g, UPCR 0.44 g/g, and UACR 216.1 mg/g, indicating improved response to therapy (Figure 2 and Table 1). Additionally, the patient experienced no adverse drug reactions during the treatment course and expressed high satisfaction with the therapeutic outcomes.

**Table 1:** Key laboratory data, drug interventions and follow-up results.

Laboratory data	Baseline	Losartan potassium		Hydroxychloroquine				Nefecon
		1 month	45 days	2.5 months	4 months	7.5 months	9 months	51 days
UACR (mg/g)	738	1569.3	1212.7	664.5	647.3	922.7	905.8	216.1
UPCR (g/g)	0.786	2.31	1.753	1.052	0.95	1.473	1.49	0.44
Scr (μmol/L)	55	61	55	57	58	58	—	60
24-h urinary protein (g)	1.42	2.68	—	0.97	0.69	1.01	—	0.68

## Discussion

IgAN was described and named by French scholar Berger in 1968. Its immunopathology is characterized by the deposition of immune complexes mainly composed of IgA in the glomerular mesangial area, with histological changes based on mesangial cell proliferation and mesangial matrix increase, also known as Berger’s disease (6). Primary IgAN occurs in patients aged 16–35 years old and is clinically characterized by microscopic hematuria, which may be accompanied by varying degrees of proteinuria, and some patients show episodic gross hematuria after mucosal infection (7). The patient in this case is a 41 year old female with clinical manifestations of microscopic hematuria and proteinuria below the level of nephrotic syndrome, without hypertension or renal dysfunction. The pathological examination results of renal tissue indicate the deposition of monotypic IgA-κ light chains in the mesangial area of the glomerulus, mesangial cell proliferation accompanied by focal capillary proliferation, and deposition of electron dense material in the mesangial area of the glomerulus. Based on the absence of skin rash and normal autoantibodies in the patient, after ruling out secondary factors, the patient was diagnosed with primary IgAN with monotypic IgA-κ deposits, and its clinical and pathological manifestations were similar to those of IgAN with polyclonal IgA deposits.

Frozen immunofluorescence (F-IF) and paraffin immunofluorescence (P-IF) are both effective methods for detecting monotypic IgA-κ deposits, but in clinical practice, simultaneous F-IF and P-IF examinations are required to determine glomerular monocyte IgA-κ deposition. F-IF has certain limitations in diagnosing monotypic IgA deposition. In F-IF, light chain proteins exist in crystalline form within cells, and the antigen sites of light chain proteins cannot be fully exposed, which may lead to incorrect light chain restriction labeling and difficulty in accurately reflecting monotypic IgA-κ deposits. Compared with F-IF, P-IF can fully expose antigen sites through antigen repair, which can more accurately reflect the deposition of glomerular light chains and further improve the accuracy and credibility of staining results (8). Therefore, if F-IF detects deposition of monotypic IgA-κ light chains in the glomerular mesangial area, further P-IF examination is required for confirmation. The F-IF and P-IF examinations of this patient in this case both indicated the deposition of monotypic IgA-κ in the glomeruli, with high reliability, providing reliable evidence for the diagnosis of primary IgAN with monotypic IgA-κ deposits. With advances in detection technology, heavy/light chain-specific immunofluorescence (HLC-IF) of intact immunoglobulins now enables more accurate identification of monotypic IgA deposition in renal tissue. In one study applying HLC-IF to renal biopsies from 12 IgAN patients showing λ light chain-restricted expression by conventional immunofluorescence, six cases demonstrated co-dominant staining for both IgA-κ and IgA-λ, effectively excluding monotypic IgA deposition (9). However, this advanced diagnostic assay has not yet been incorporated into our pathology center’s testing repertoire. Previous studies have confirmed that IgA includes two subtypes, IgA1 and IgA2, but only IgA1 is involved in the pathogenesis of IgAN, while IgA2, due to the lack of hinge regions and different glycosylation patterns, is almost not involved in the pathogenesis of IgAN (10). Therefore, the possibility of glomerular deposition in this patient is monotypic IgA1-κ.

In recent years, there has been an increasing number of reports on IgAN with single light chain IgA deposition in immunofluorescence, mostly described as monotypic IgA deposits (3), monoisomorphic IgA deposits (11), and light chain restriction expression (12). Although the descriptions are different, their essence is a special type of IgAN. According to statistics, 0–42% of primary IgAN’s mesangial area shows deposition of monotypic IgA, mainly IgA-λ, with only a few IgA-κ (2). Lai et al. (13) reported the pathological conditions of 45 patients with primary IgAN, of whom 19 patients (42%) had monotypic IgA deposition, of which only one case had monotypic κ light chain deposition. Nagae et al. (2) analyzed 65 cases of IgAN and found that six patients (9.2%) had monotypic IgA deposition, of which only one case had monotypic κ light chain deposition. Katafuchi et al. (3) summarized the pathological conditions of 526 cases of IgAN and found that there were 33 cases (6.3%) of monotypic IgA deposition, including 12 cases of monotypic κ light chain deposition. In terms of clinical and pathological manifestations, the above studies have found no significant difference between IgAN patients with monotypic IgA deposits and those with polyclonal IgA deposits. No monoclonal IgA was detected in the serum, and the blood free light chain ratio was also normal. During follow-up, no patients progressed to hematological malignancies. However, most previous studies have only summarized the clinical and pathological characteristics of IgAN with monotypic IgA-λ deposits, and there are few reports on the clinical and pathological features of IgAN with monotypic IgA-κ deposits. In this case, we report a case of IgAN with monotypic IgA-κ deposits, whose clinical and pathological manifestations were similar to those of IgAN with polyclonal IgA deposits, and no monoclonal IgA was detected in serum and urine. In addition, unlike previous studies (2, 3, 13–15), this case underwent further immunoelectron microscopy examination, and no abnormal deposition of monoclonal light chains was found, providing further evidence for the diagnosis of this case as IgAN with monotypic IgA-κ deposits and deepening our understanding of the clinical and pathological characteristics of IgAN with monotypic IgA-κ deposits. The above evidence suggests that IgAN with monotypic IgA-κ deposits may be a special pathological subtype of IgAN with polyclonal IgA deposits (Table 2).

**Table 2:** Summary of the previously studies reported IgAN with monotypic IgA deposits.

Studies	Monotypic IgA deposits	Monotypic kappa	Monotypic lambda	Clinicopathology	Hematological disorder
Nagae et al. (2)	6	1 (1.5%)	5 (7.7%)	The clinicopathology of monotypic IgA-λ deposits were similar to the patients with polyclonal IgA deposition	No one
Katafuchi et al. (3)	33	12 (2.3%)	21 (4.0%)	No distinct clinicopathology differences between patients with monotypic IgA deposition and those with polytypic one	No one
Lai et al. (13)	19	1 (2.2%)	18 (40%)	The patients with predominant lambda light chain had lower serum creatinine concentration and higher creatinine clearance, but these data failed to reach statistical significance	Not examined
Orfila et al. (14)	11	5 (13.5%)	6 (16.2%)	Not described	Not examined
Uppin et al. (15)	22	9 (15.5%)	13 (22.4%)	Not described	Not examined

The current treatment for primary IgAN still relies on RAS inhibitors as the base drug. Previous studies have confirmed that the treatment regimen of RAS inhibitors combined with hydroxychloroquine can significantly reduce urinary protein in IgAN patients (16). The 2021 KDIGO guidelines also recommend the use of hydroxychloroquine to treat Chinese IgAN patients whose urinary protein has not yet improved after optimized supportive therapy (17). In this case, since the patient’s proteinuria did not show significant reduction with losartan potassium monotherapy, the patient was then treated with the maximum tolerated dose of RAS inhibitors combined with hydroxychloroquine. After 11 months of follow-up, the patient’s urinary protein decreased compared to before treatment, but still remained >1 g/24 h. In the pathogenesis of IgAN, the production of galactose deficient IgA1 (Gd IgA1) is a key factor leading to disease occurrence (18). This Gd-IgA1 molecule is believed to primarily originate from mucosa associated lymphoid tissue (19) and is produced under the stimulation of infection associated inflammatory mediators (10). Studies have shown that gut dysbiosis can trigger mucosal immune dysfunction, which serves as a potential driver for the formation of nephrotoxic immune complexes specific to IgA nephropathy (20). Nefecon is a targeted release formulation of the novel glucocorticoid budesonide, which specifically acts on the intestinal mucosal immune system, inhibiting the activation of B cells that produce Gd-IgA1, reducing the deposition of Gd-IgA1 immune complexes in the kidneys, thereby reducing UPCR and stabilizing eGFR levels. In addition, it can also reduce the risk of systemic immune suppression caused by traditional hormones. The drug has been approved for the treatment of IgAN (21). In this case, despite 9 months of combination therapy with losartan potassium and hydroxychloroquine, the patient’s proteinuria levels remained persistently elevated, we therefore initiated adjunctive treatment with Nefecon, and after more than 1 month, the patient’s urinary protein decreased to 0.68 g/24 h, with no adverse drug reactions observed during the treatment course. Therefore, the traditional method of treating IgAN with polyclonal IgA deposits is equally effective in treating IgAN with monotypic IgA-κ deposits and IgAN with monotypic IgA-κ deposits is more sensitive to drug response to Nefecon (targeting intestinal mucosal B cells). This result suggests that mucosal immune dysfunction may also be the core pathogenesis of IgAN with monotypic IgA-κ deposits.

It is worth noting that there is a certain similarity in pathological manifestations between IgAN with IgA-κ deposits and proliferative glomerulonephritis with monoclonal IgA deposits (IgA-PGNMID), but the latter has a risk of progressing to hematological malignancies, a worse prognosis, and requires targeted B cell/plasma cell therapy. Therefore, in clinical practice, it is necessary to distinguish between IgAN with monotypic IgA-κ deposits and IgA-PGNMID. PGNMID was first reported by Nasr et al. (22), and is characterized by monoclonal gammopathy of renal significance (MGRS). MGRS is most frequently observed in individuals over 50 years old (65%). The predominant subtype is IgG3κ (53%), with histopathological changes primarily manifesting as membranoproliferative glomerulonephritis (57%) and endocapillary proliferative glomerulonephritis (35%). Additionally, 10% of patients exhibit decreased serum complement levels (23). Compared with IgG3κ-PGNMID, IgA-PGNMID is relatively rare, but their pathological features are similar (24). Unlike the source of IgA-κ, the deposition of monoclonal IgA in IgA-PGNMID glomeruli is caused by excessive amplification of bone marrow monoclonal B lymphocytes or plasma cells, leading to excessive secretion of monoclonal IgA. The latter not only has homologous immunoglobulin heavy chains and subtypes, but also homologous light chains, as well as the same heavy chain variable region (VH) and light chain variable region (VL). However, due to the limitations of ordinary laboratory conditions, it is difficult to carry out routine sequencing of amino acids in the variable region. Therefore, the deposition of monoclonal immunoglobulin in the kidneys combine with the detection of corresponding monoclonal immunoglobulin in blood or urine are strong evidence for the diagnosis of PGNMID (11). In this case, no monoclonal IgA-κ was detected in the patient’s serum and urine before renal puncture and during follow-up. The blood free light chain ratio was also normal. Renal pathological examination showed mesangial cell proliferation as the main cause, accompanied by focal capillary proliferation in the glomerulus. No pathological changes similar to membrane proliferative glomerulonephritis were found in PGNMID. Electron microscopy examination did not reveal the deposition of special structural substances, and immunoelectron microscopy labeling did not reveal exact monoclonal light chain abnormal deposition. Therefore, in this case, the existing evidence does not support the diagnosis of IgA-PGNMID. However, it should be noted that the detection rate of monoclonal immunoglobulin in the blood and urine of IgA-PGNMID patients is very low, and one-third of cases may be misdiagnosed as IgAN in the early stages of the disease. Vignon et al. (24) used conventional techniques to detect 14 patients diagnosed with IgA-PGNMID, and only found monoclonal IgA in the blood or urine of five patients (36%) that matched renal tissue deposition. More sensitive tests (such as western blotting or bone marrow molecular studies) were used to detect the remaining nine patients, and it was found that two patients had low levels of monoclonal IgA in their serum, and two patients had bone marrow small B cell clone populations in their serum. The above research suggests that conventional serum and urine immunofixation electrophoresis may be difficult to detect monoclonal immunoglobulins, and more sensitive tests are needed to improve the detection rate of monoclonal immunoglobulins. In this case, the patient has only been followed up for 12 months, and further follow-up time is needed to closely monitor hematological indicators. If necessary, more sensitive techniques such as immunoblotting or bone marrow molecular studies should be used to exclude the diagnosis of IgA-PGNMID.

Studies have shown that the detection rate of nephrotoxic monoclonal immunoglobulin in serum or urine, as well as abnormal bone marrow B-cell clones, is only ∼30% (25). Therefore, monitoring for the possible emergence of paraproteinemias may not be useful in differentiating between these two renal pathologies. However, in elderly patients presenting with pathological features such as membranoproliferative-like changes accompanied by hypocomplementemia, or electron microscopic evidence of distinctive structured deposits, along with a pathological diagnosis of IgAN with monotypic IgA deposits, a high suspicion for IgA-PGNMID should be maintained. Some researchers also suggest that KM55 monoclonal antibody can serve as an effective method for distinguishing between IgAN with monotypic IgA-κ deposits and IgA-PGNMID (26, 27). KM55 is a Gd-IgA1 specific monoclonal antibody, which is a novel method for detecting Gd-IgA1 in human serum and mesangial region. Suzuki et al. (27) used KM55 detection method to detect the serum Gd-IgA1 concentration and glomerular Gd-IgA1 expression level in patients with primary IgAN, the results showed that 27.6% of patients had elevated serum Gd-IgA1 levels and positive glomerular Gd-IgA1, and Gd-IgA1 was mainly located in the mesangial area and co localized with IgA, while similar phenomena were not found in other secondary IgAN. Zhang et al. (28) used KM55 staining method to detect 2 cases of IgA-PGNMID and 10 cases of IgAN patients with monotypic IgA-κ deposits, the results showed that KM55 staining in the glomeruli of the former was negative, while the latter was positive. The above research results suggest that KM55 can serve as an important basis for distinguishing between monotype IgA deposition IgAN and IgA-PGNMID.

There have been numerous reported cases of monoclonal IgA-κ deposition in renal tissue. Li et al. (29) described a case of a 49-year-old woman with glomerular IgA-κ positive deposits. Electron microscopy revealed deposition of disordered fibrous substances, approximately 10–20 nm in diameter, in the subepithelial layers, basement membrane, and mesangial region. Immunoelectron microscopy demonstrated deposition of fibrous materials measuring about 10–20 nm in diameter with disordered arrangement. The κ light chain showed positivity along the fibrous substances, while the λ light chain was negative, leading to a diagnosis of fibrillary glomerulonephritis. Kaneko et al. (30) reported a case of mesangial proliferative glomerulonephritis with mIgA (IgA-κ) deposition. This case showed monoclonal IgA-κ detected by serum immunofixation electrophoresis and was diagnosed as monoclonal immunoglobulin deposition disease (MIDD) associated with monoclonal IgA. In our present case, immunoelectron microscopy revealed no abnormal monoclonal light chain deposition, no elevated monoclonal immunoglobulin was detected in serum or urine, and the patient demonstrated good response to treatment with targeted-release formulation of the new glucocorticoid budesonide. Therefore, the diagnosis is more consistent with primary IgAN.

The mechanism underlying the deposition of monotypic IgA-κ is currently unclear. Previous studies have shown that monotypic IgA deposition in IgAN is more common with IgA-λ, possibly due to the abundance of B cells producing Gd-IgA1-λ in mucosa associated lymphoid tissue (4), as well as the stronger affinity of negatively charged λ light chains for mesangial tissue (5). However, the mechanism of monotypic IgA-κ deposition is still unclear. Zachova et al. (31) found that although Gd-IgA1^+^ cells in IgAN patients preferentially express lambda light chains (68%), total IgA^+^ cells preferentially express κ light chains (65%). Therefore, increased expression of κ light chains may be a potential factor causing IgA-κ deposition in the kidneys. In addition, the decreased ability of the body to clear the κ light chain may also be the reason for the deposition of the κ light chain in the kidneys, leading to the deposition of monomeric IgA-κ in IgAN. A scholar reported a case of a 60-year-old male patient with alcoholic cirrhosis, the renal pathological examination showed the presence of “monoclonal” IgA-κ deposition in the mesangial area and capillary wall of the glomerulus, but there were no abnormalities in serum, urine protein electrophoresis and serum free light chain ratio, indicating insufficient evidence for the diagnosis of myeloproliferative disease (32). Therefore, reduced clearance of IgA-κ by the liver may also lead to the deposition of monomeric IgA-κ in the kidneys. However, the exact mechanism that causes IgA-κ deposition in the kidneys needs further investigation.

## Conclusion

In conclusion, IgAN with monotypic IgA-κ deposits may be a special immunopathological manifestation of IgAN with polyclonal IgA deposits. The clinical and pathological manifestations and prognosis of the two are similar. However, as this case report involves only a single patient with relatively short follow-up duration, large-scale and randomized controlled studies are still needed to further clarify the association between the two. The mechanism of the deposition of IgAN by monotypic IgA-κ is currently unclear. Increased expression of κ light chain or reduced clearance of IgA-κ by the body may be the cause of the deposition of IgAN by monotypic IgA-κ. In addition, patients with IgAN accompanied by monotypic IgA-κ deposition should be monitored for possible IgA-PGNMID. The detection of monoclonal immunoglobulin in blood or urine that matches the deposits found in renal tissue provides strong evidence for a diagnosis of PGNMID, and early differentiation and treatment of the two are crucial for improving patient prognosis.

## Data Availability

The original contributions presented in the study are included in the article/supplementary material, further inquiries can be directed to the corresponding author.
